# Alveolar Type II Cells Possess the Capability of Initiating Lung Tumor Development

**DOI:** 10.1371/journal.pone.0053817

**Published:** 2012-12-20

**Authors:** Chuwen Lin, Hai Song, Cecilia Huang, Erica Yao, Rhodora Gacayan, Shan-Mei Xu, Pao-Tien Chuang

**Affiliations:** Cardiovascular Research Institute, University of California San Francisco, San Francisco, California, United States of America; University of Pennsylvania School of Medicine, United States of America

## Abstract

Identifying cells of tumor origin is a fundamental question in tumor biology. Answers to this central question will not only advance our understanding of tumor initiation and progression but also have important therapeutic implications. In this study, we aimed to uncover the cells of origin of lung adenocarcinoma, a major subtype of non-small cell lung cancer. To this end, we developed new mouse models of lung adenocarcinoma that enabled selective manipulation of gene activity in surfactant associated protein C (SPC)-expressing cells, including alveolar type II cells and bronchioalveolar stem cells (BASCs) that reside at the bronchioalveolar duct junction (BADJ). Our findings showed that activation of oncogenic *Kras* alone or in combination with the removal of the tumor suppressor *p53* in SPC^+^ cells resulted in development of alveolar tumors. Similarly, sustained EGF signaling in SPC^+^ cells led to alveolar tumors. By contrast, BASCs failed to proliferate or produce tumors under these conditions. Importantly, in a mouse strain in which *Kras/p53* activity was selectively altered in type II cells but not BASCs, alveolar tumors developed while BADJs retained normal architecture. These results confirm and extend previous findings and support a model in which lung adenocarcinoma can initiate in alveolar type II cells. Our results establish the foundation for elucidating the molecular mechanisms by which lung cancer initiates and progresses in a specific lung cell type.

## Introduction

Non-small cell lung cancer (NSCLC) accounts for ∼85% of lung cancers and most patients present with advanced disease [1]. Despite the investment of an enormous amount of effort and resources in the past few decades, the treatment options remain very limited for advanced or recurrent NSCLC [2], [3]. For inoperable tumors, the standard treatment consists of various combinations of chemotherapy, radiotherapy and targeted therapies [4]. Improvements in prognosis have been modest, with an overall 5-year survival rate of only 10–15%. This is in sharp contrast to the drastically improved 5-year survival rates in patients afflicted by many other types of solid tumors such as breast or prostate cancer. These achievements are due to early diagnosis and effective treatment. This highlights an urgent need for basic research to identify the cells from which lung cancer originates and uncover the molecular basis of tumor development in order to design new therapies [5]–[8].

Based on mutation profiles identified in human NSCLC [9], several mouse models of NSCLC have been created [10]–[15]. A widely used model combines *p53* ablation with *Kras* activation in the mouse lung epithelium [16]–[18]. These mice developed lung adenocarcinoma (the major subtype of NSCLC) [19] that recapitulates critical aspects of human NSCLC. However, this approach relies on inactivation of *p53* and activation of *Kras* in the lung epithelium globally [20]. It does not allow for identifying the cells of origin of NSCLC or isolating a pure population of tumor cells at distinct stages of tumor development for genome-wide analysis. Addressing these issues requires additional mouse models in which oncogenes and tumor suppressors can be selectively manipulated in a given cell population to test their role in tumor development.

Another important mouse model of NSCLC is based on the finding that a fraction of patients whose tumors harbor mutations in the gene encoding the epidermal growth factor receptor (EGFR) [21], [22] are responsive to tyrosine kinase inhibitors (TKIs) that block EGF signaling. Mice carrying various EGFR mutations found in human lung cancer develop lung tumors [23], [24], providing an appropriate animal model for studying NSCLC development and drug resistance. Likewise, the cells of origin of lung tumors in these systems have not been rigorously addressed due to the lack of tools that enable selective manipulation of gene activity in a particular lung cell type.

Investigating the cells of origin of lung cancers will provide new insight into the molecular basis of tumor initiation and progression. It is possible that human lung cancers that originate from different cell types possess characteristic mutation profiles and behaviors. Distinct cell types in the airway and alveoli [25] that fulfill requisite physiological functions of the lung are also candidates as the initiating cells of adenocarcinoma [26]. Secretory non-ciliated Clara cells in the bronchioles are interspersed with ciliated cells and neuroendocrine cells, whereas type I and type II cells line the gas-exchange epithelium of the alveoli. Bronchioalveolar stem cells (BASCs) [27] express markers for Clara cells (CC10) and type II cells (SPC) and are located at the bronchioalveolar duct junction (BADJ) where terminal bronchioles connect with the alveoli. The physiological function of BASCs remains unclear [28]. Clara cells, BASCs and type II cells possess proliferative potential in various conditions and could potentially accumulate mutations in these processes to initiate tumor development. Nevertheless, it remains uncertain whether tumor development can also begin in non-proliferative, differentiated cell types or transformation is largely confined to cells with progenitor potential. In this study, we utilize mouse lines that allow the manipulation of gene activities in alveolar type II cells and BASCs to test these ideas. Our results support a model in which lung adenocarcinoma can initiate in type II cells. These studies provide the basis for further investigation to uncover the molecular mechanisms of tumor development and progression.

## Results

### Inducible Expression of CreER Recombinase or Activation of rtTA Under the Control of the *SPC* Promoter Allows the Manipulation of Gene Activity in SPC^+^ cells Including Alveolar Type II and BASC cells

**Figure 1:**
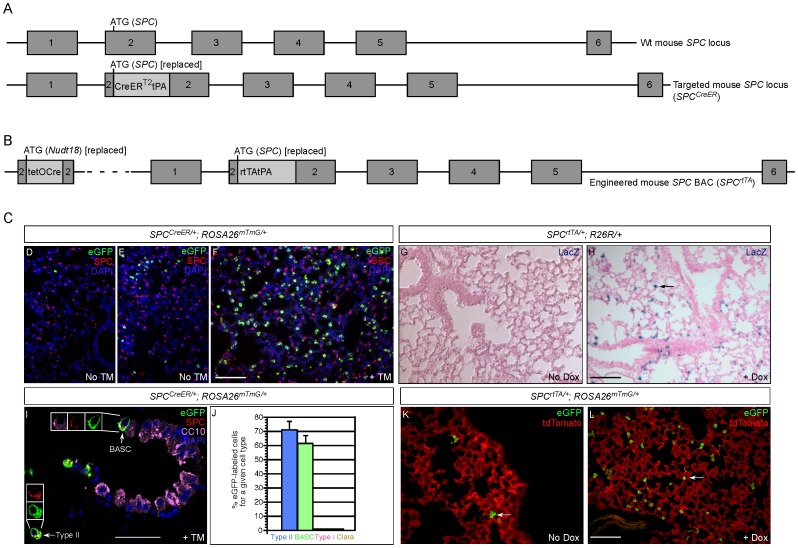
*SPC^CreER^* and *SPC^rtTA^* mice allow manipulation of gene activities in alveolar type II cells. (A) Schematic diagram depicting gene targeting of a CreERT2tPA cassette at the translational start (ATG) of the mouse *SPC* genomic locus (exons are numbered). tPA contains three copies of the polyadenylation signal (PA). The resulting allele is designated *SPC^CreER^* in this study. Insertion of CreER disrupts the production of *SPC* mRNA but heterozygous *SPC^CreER^* mice are viable and exhibit no obvious phenotypes, similar to previous reports [47]. (B) Schematic diagram illustrating the introduction of an rtTAtPA cassette at the translational start (ATG) of the mouse *SPC* genomic locus contained within a BAC. The engineered BAC was subsequently modified to introduce a tetO-Cre cassette at the translational start (ATG) of the *Nudt18* genomic locus located approximately 57 kb upstream of the translational start of *SPC*. The transgenic mouse line carrying the modified BAC is designated *SPC^rtTA^* in this study. (C) Immunostaining and immunohistochemistry to assess the specificity and efficiency of *SPC^CreER^* and *SPC^rtTA^* mouse lines. (D–F) Immunostaining of lung sections from adult *SPC^CreER/+^*; *ROSA26^mTmG/+^* mice. Without tamoxifen (TM) injection, low levels of CreER activity in alveolar type II cells (identified by anti-SPC) resulted in eGFP expression (detected by anti-GFP antibodies) from the *ROSA26^mTmG^* allele by removing sequences that block its expression. The levels of leaky CreER expression in alveolar type II cells seem to vary from animal to animal (D, E) and could even differ between lung lobes of a single uninjected animal. TM administration led to a dramatic increase in the number of eGFP-labeled type II cells (F), indicating efficient activation of CreER by TM. In addition to type II cells, SPC^+^CC10^+^ bronchioalveolar stem cells (BASCs) located at the bronchioalveolar duct junction (BADJ) were also efficiently labeled by eGFP upon TM injection (I). The insets in (I) show individual images of eGFP/SPC/CC10 immunostaining of the same BASC (arrow) or eGFP/SPC immunostaining of the same type II cell (arrow). The percentage of distinct cell types labeled by eGFP upon TM administration is summarized in (J). At least five mice and more than three sections per mouse were examined. Since BASCs are less prevalent than type II, type I and Clara cells, all BASCs present on a given section were counted. The proportion of lineage-labeled cells was scored as follows: type II cell (ratio of SPC^+^eGFP^+^/eGFP^+^); BASC (ratio of SPC^+^CC10^+^eGFP^+^/SPC^+^CC10^+^ at BADJs); type I cell (ratio of T1α^+^eGFP^+^/eGFP^+^); and Clara cell (ratio of CC10^+^eGFP^+^SPC^−/^eGFP^+^). (H) β-galactosidase (LacZ) staining of lung sections from adult *SPC^rtTA/+^*; *R26R/+* mice fed with dox (doxycycline) chow. LacZ^+^ type II cells (arrow) were widespread, indicating efficient activation of the reporter by tetO-regulated Cre that is expressed when rtTA is bound by dox. Similar conclusions were reached when analysis was performed on lung sections from *SPC^rtTA/+^*; *ROSA26^mTmG/+^* mice fed with dox chow (L; arrow points to a type II cell). In the absence of dox, low levels of rtTA activity led to labeling of some type II cells (*e.g.*, arrow in K), similar to a low degree of leakiness observed in *SPC^CreER^* mice. Scale bar = 100 µm for panels in each row except (I), which is 50 µm.

Proliferation of alveolar type II cells significantly increases following lung injury. Type II cells also have the potential to generate type I cells, which are responsible for gas exchange in the alveoli [25]. These progenitor-like properties render type II cells a prime candidate for initiating lung adenocarcinoma. To regulate gene activity in type II cells, we generated two mouse lines by (1) introducing CreER into the endogenous mouse *SPC* (*Sftpc*; *surfactant associated protein C*) locus via gene targeting [29] and (2) introducing rtTA into an *SPC*-containing bacterial artificial chromosome (BAC) for producing transgenic mice. This is based on the finding that SPC constitutes the major peptide in type II cells and is not present in other lung cell types [30] except the SPC^+^CC10^+^ BASCs at the BADJ. The resulting mouse lines are designated *SPC^CreER^* (Fig. 1A) and *SPC^rtTA^* (Fig. 1B) respectively in this study and they enable control of gene activity in SPC-expressing type II cells and BASCs.

CreER encodes a fusion protein of Cre recombinase and estrogen receptor (ER) and is activated by tamoxifen (TM) [31]. Thus, tamoxifen administration to *SPC^CreER^* mice provides an efficient way to control Cre activity in a temporal- and spatial-specific manner. For example, tamoxifen injection into adult *SPC^CreER^* mice should allow exclusive Cre expression in SPC-expressing cells, including type II cells and BASCs, after lung development is completed. Selective, inducible Cre expression in conjunction with Cre-lox technology offers a unique opportunity to regulate gene activity in type II cells and BASCs. We tested the efficiency and specificity of CreER expression in *SPC^CreER^* mice by breeding *SPC^CreER^* mice with several reporter mouse lines, including *R26R* [32] and *ROSA26^mTmG^* [33]. Cre activation would result in eGFP expression from the *ROSA26^mTmG^* allele by removing sequences that block its expression. For example, we found that daily injection of tamoxifen (0.25 mg/g body weight) for four to six times into *SPC^CreER/+^; ROSA26^mTmG/+^* adult mice led to efficient expression of CreER, resulting in eGFP expression in SPC^+^ type II cells (Fig. 1F) and BASCs (Fig. 1I). A low level of CreER activity without TM injection in type II cells was noted (Fig. 1D, 1E) and leaky expression of CreER appears to vary from animal to animal. The specificity of CreER expression in type II cells was demonstrated by the observation that few SPC(–) cells were labeled by eGFP expression in *SPC^CreER/+^; ROSA26^mTmG/+^* lungs (Fig. 1J). These findings indicate that CreER expression from *SPC^CreER^* mice is specific and sufficient to permit manipulating gene activity in SPC-expressing cells.

The tetracycline inducible rtTA (Tet-On) system consists of two major elements: rtTA and TRE (tet response element; denoted as tetO) [34]. In the absence of doxycycline (dox), rtTA does not bind or binds weakly to TRE and the gene placed under TRE control is not transcribed. However, in the presence of the inducing agent dox, rtTA binds to TRE, which in turns activates transcription of TRE-controlled genes. We introduced rtTA into the *SPC* locus and tetO-Cre into the *Nudt18* locus contained within a single BAC clone (designated *SPC^rtTA^* for simplicity) and produced the transgenic mouse line *SPC^rtTA^* by pronuclear injection [35] (Fig. 1B). tetO-Cre was introduced into a separate genomic locus to avoid any potential interference with rtTA. When dox food was used continuously for *SPC^rtTA/+^; R26R/+* or *SPC^rtTA/+^; ROSA26^mTmG/+^* mice, extensive labeling of type II cells was observed (Fig. 1H, 1L) in contrast to the spotty labeling in *SPC^rtTA/+^; R26R/+* or *SPC^rtTA/+^; ROSA26^mTmG/+^* mice without dox (Fig. 1G, 1K). This suggests that rtTA expression from *SPC^rtTA/+^* mice should enable manipulating gene activity in SPC^+^ cells, including all genes that are placed under TRE (tetO) control.

### Activating *Kras* Alone or in Combination with *p53* Removal in Murine SPC^+^ cells Led to Non-small Cell Lung Cancer (NSCLC) Development in Alveoli

Loss of *p53* and activated *Kras* mutations are frequently found in human NSCLC [9]. This prompted several groups to develop a mouse model of NSCLC through global inactivation of *p53* and activation of *Kras* (*G12D* mutation) in the lung epithelium, which was achieved via intrabronchial injection of adenoviral Cre [16]–[18]. This mouse model of NSCLC recapitulates critical aspects of human NSCLC, validating the use of mouse models for understanding human NSCLC [12]. However, this approach carries several important caveats, including the side effects of anti-inflammatory agents used to suppress adenovirus-induced inflammation, the inconsistent delivery and expression of adenoviral Cre that could result in variable tumor development and perhaps most importantly, the inability to pinpoint the cell types that initiate transformation.

To address these issues, we employed the *SPC^CreER^* mouse line to activate *Kras* and inactivate *p53* in SPC^+^ cells. We took advantage of the widely used *Kras^LSL-G12D^* and *p53^f^* alleles. *Kras^LSL-G12D^* is converted into activated *Kras^G12D^* by Cre, while the conditional *p53^f^* allele is converted into a null allele in the presence of Cre. This offers a precise genetic system to manipulate *p53* and *Kras* exclusively in SPC^+^ cells. We bred mice to produce *SPC^CreER/+^; Kras^LSL-G12D/+^* and *SPC^CreER/+^; p53^f/f^; Kras^LSL-G12D/+^* mice and administered tamoxifen to these adult animals. Lungs were collected at different time points after TM injection for analysis.

**Figure 2:**
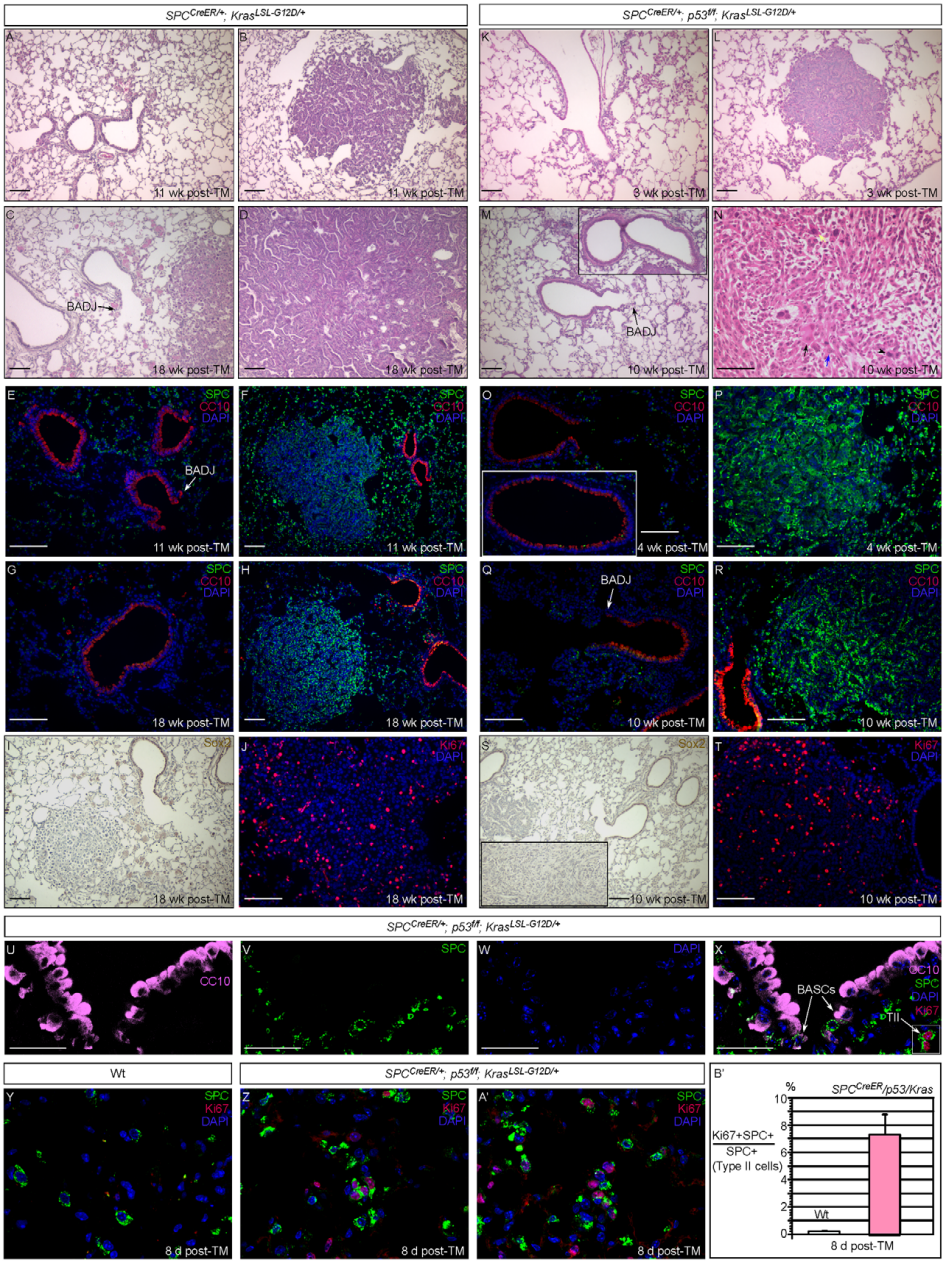
Induction of alveolar lung tumors in mice with activated *Kras* alone or in combination with loss of *p53* in SPC-expressing cells. (A–D) Histology of lung sections from *SPC^CreER/+^; Kras^LSL-G12D/+^* adult mice injected with tamoxifen (TM) to activate *Kras*. These mice developed alveolar tumors while no abnormalities were detected in the airway or bronchioalveolar duct junction (BADJ). Increase in average tumor size with time was observed. (E–J) Immunostaining of lung sections from *SPC^CreER/+^; Kras^LSL-G12D/+^* adult mice injected with TM to activate *Kras*. Alveolar tumors shown in (F, H, I) expressed SPC but not CC10 or Sox2. Airways and BADJs displayed normal architecture. Tumor cells were highly proliferative as assessed by Ki67 staining in (J), the entire field of which consists of tumor cells. (K–N) Histology of lung sections from *SPC^CreER/+^; p53^f/f^; Kras^LSL-G12D/+^* adult mice injected with TM to activate *Kras* and remove *p53* simultaneously. Similar to *SPC^CreER/+^; Kras^LSL-G12D/+^* mice, *SPC^CreER/+^; p53^f/f^; Kras^LSL-G12D/+^* mice developed alveolar tumors while the airway or BADJ appeared normal. The inset in (M) shows the airway. Compared to tumors in *SPC^CreER/+^; Kras^LSL-G12D/+^* mice at the same age, tumor growth was in general accelerated and cell morphology indicates higher-grade tumors in *SPC^CreER/+^; p53^f/f^; Kras^LSL-G12D/+^* mice, for instance, by the presence of prominent nucleoli (blue arrow), enlarged pleomorphic nuclei (arrowhead), aberrant mitosis (yellow arrow) and tumor giant cells (black arrow) in (N). (O–T) Immunostaining of lung sections from *SPC^CreER/+^; p53^f/f^; Kras^LSL-G12D/+^* adult mice with activated *Kras* and loss of *p53*. Similarly, alveolar tumors (P, R, S) expressed SPC but not CC10 or Sox2. The inset in (O) displays the airway and the inset in (S) showed an alveolar tumor. These tumor cells were highly proliferative as assessed by Ki67 staining (T), the entire field of which consists of tumor cells. (U–X) Immunostaining of lung sections from *SPC^CreER/+^; p53^f/f^; Kras^LSL-G12D/+^* adult mice with activated *Kras* and loss of *p53*. BASCs (SPC^+^CC10^+^) at the BADJs rarely proliferated whereas extensive type II cell (TII) (SPC^+^) proliferation was observed. Similar results were obtained in *SPC^CreER/+^; Kras^LSL-G12D/+^* adult mice. (Y–A’) Immunostaining of lung sections from wild-type (Wt) and *SPC^CreER/+^; p53^f/f^; Kras^LSL-G12D/+^* adult mice at eight days post-TM injection. Significant proliferation of type II cells (SPC^+^) was apparent in *SPC^CreER/+^; p53^f/f^; Kras^LSL-G12D/+^* adult mice. (B’) Quantification of proliferating type II cells at eight days after TM administration. While BASCs have been shown to expand under various experimental conditions, the use of adenoviruses to achieve global perturbation of gene activity does not allow for identification of cells of tumor origin. It is possible that cells other than BASCs are the primary targets of genetic perturbations and these cells express both SPC and CC10 markers as a result of genetic perturbation. Scale bar = 100 µm for each panel except (U–X), which is 50 µm.

By 18 weeks after TM injection, *SPC^CreER/+^; Kras^LSL-G12D/+^* mice developed numerous tumors in the alveoli, largely adenomas and a small number of cells with features of malignant transformation (Fig. 2B, 2D), while the airways and BADJs retained normal architecture (Fig. 2A, 2C, 2E, 2G). These tumors stained positive for SPC (negative for CC10 and Sox2) (Fig. 2F, 2H, 2I) and were morphologically similar to those produced in a lung cancer model in which adenoviral Cre was used to induce *Kras^G12D^* expression [16]–[18]. Tumor cells exhibited high proliferation (Fig. 2J). This phenotype is completely penetrant, follows a defined time sequence and does not appear to be affected by genetic background. We noted that a significant fraction of tumors was found in close proximity to the airway or BADJ or the surface of the lung. Interestingly, the BADJ itself did not contain hyperplastic lesions or tumors.

Compared to *SPC^CreER/+^; Kras^LSL-G12D/+^* mice at the same stage, *SPC^CreER/+^; p53^f/f^; Kras^LSL-G12D/+^* mice exhibited a more severe phenotype in terms of tumor size and tumor growth and grade as a result of *p53* elimination in addition to *Kras* activation. At 3–4 weeks after TM injection, several small adenomas of papillary nature could already be found in the alveoli of *SPC^CreER/+^; p53^f/f^; Kras^LSL-G12D/+^* mice (Fig. 2L). These lesions expressed SPC but not CC10 (Fig. 2P) or Sox2 (not shown). At 10 weeks after TM injection, large tumors were widespread in the alveoli and they exhibited features characteristic of higher grade lung tumors such as the presence of enlarged pleomorphic nuclei, aberrant mitosis and tumor giant cells (Fig. 2N). Tumor cells were also highly proliferative and invasive (Fig. 2T). In fact, many *SPC^CreER/+^; p53^f/f^; Kras^LSL-G12D/+^* mice did not survive beyond this stage due to the heavy burden of lung tumors. In mice that did survive to 13 weeks after TM injection, their lungs contained adenocarcinoma in the alveoli consisting of cells with features of malignant transformation surrounded by stromal cells. Similarly, all tumors examined stained positive for SPC (Fig. 2R) but not CC10 (Fig. 2R) or Sox2 (Fig. 2S). Since *Sox2* is expressed in the conducting airway (including cells at the BADJs) and not SPC^+^ cells in the alveoli, lack of *Sox2* expression in lung tumors would be consistent with a tumor origin from type II cells. Surprisingly, BADJs appeared normal in *SPC^CreER/+^; p53^f/f^; Kras^LSL-G12D/+^* mice at all time points examined (Fig. 2K, 2M, 2O, 2Q) even though *SPC^CreER^* was expressed in a significant fraction of BASCs in BADJs (Fig. 1I, 1J). We performed a careful analysis of BASC proliferation in both *SPC^CreER/+^; Kras^LSL-G12D/+^* and *SPC^CreER/+^; p53^f/f^; Kras^LSL-G12D/+^* mice injected with tamoxifen. While extensive type II cell proliferation was observed (Fig. 2U–X), proliferating BASCs were rarely found (Fig. 2U–X) when *Kras* was activated either alone or in conjunction with *p53* removal. BASC proliferation was assessed at eight days, three weeks, six weeks, ten weeks and thirteen weeks after TM injection and similar results were obtained. Interestingly, proliferation of SPC^+^ type II cells that carry *p53* and *Kras* mutations can be detected in just a few days after TM administration (Fig. 2Y–Z and 2A’–B’). Taken together, our findings suggest that alveolar tumors induced by *Kras/p53* perturbation were derived from alveolar type II cells and not BASCs. They support a model in which SPC-expressing type II cells proliferate in response to oncogene activation/tumor suppressor removal and initiate tumor development.

### Leaky *SPC^CreER^* Expression without Tamoxifen Injection was Found in Some Alveolar Type II Cells and Not BASCs in Certain *SPC^CreER/+^; p53^f/f^; Kras^LSL-G12D/+^* mice, Resulting in Proliferation and Transformation of Alveolar Type II and Not BASC cells

**Figure 3:**
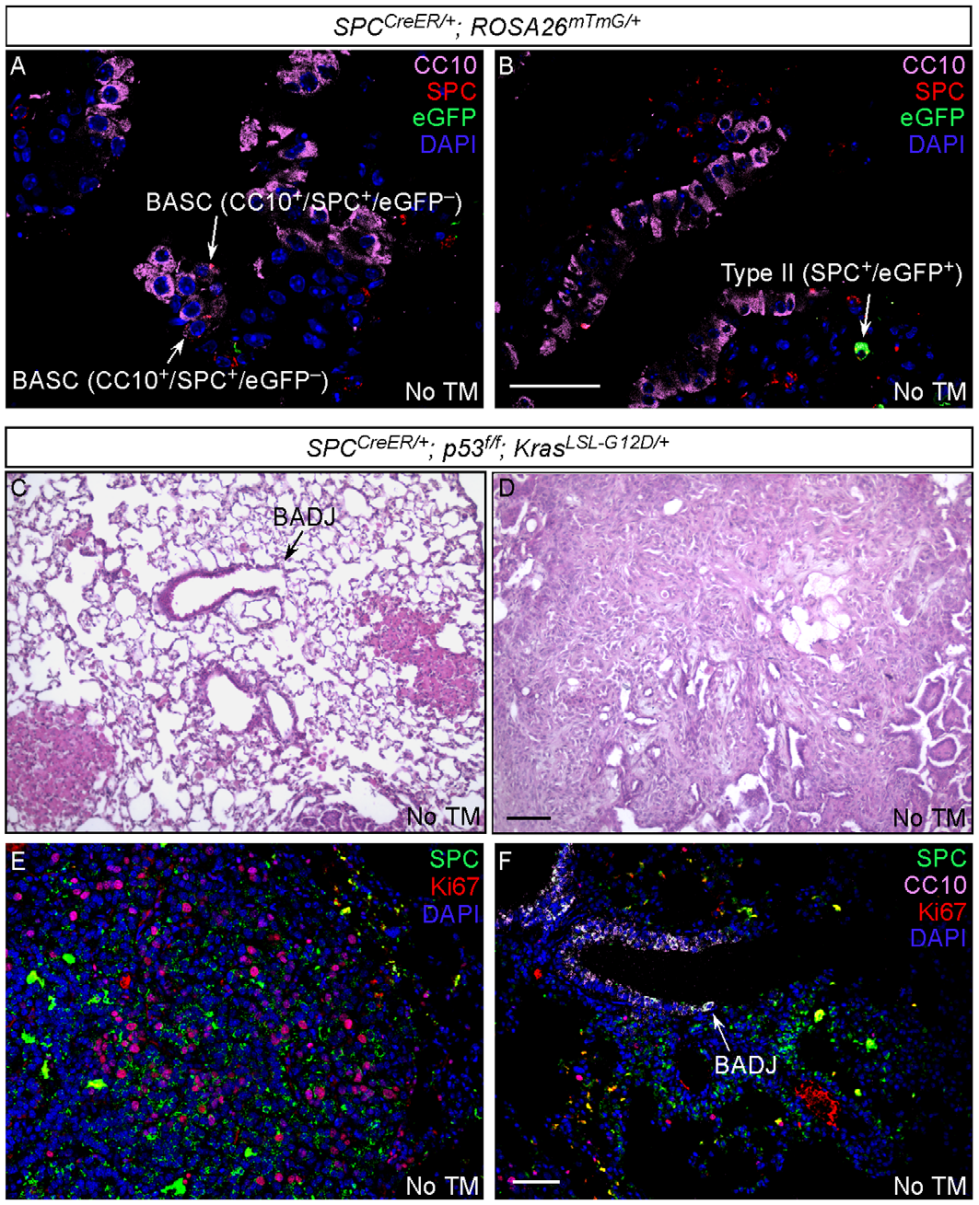
Leaky expression of *SPC^CreER^* in alveolar type II cells and not BASCs is associated with alveolar tumors while BADJs retain normal architecture. (A–F) Histology and immunostaining of lung sections from adult *SPC^CreER/+^; ROSA26^mTmG/+^* mice without tamoxifen injection. In *SPC^CreER/+^; ROSA26^mTmG/+^* mice that displayed leaky Cre activity without tamoxifen injection, leaky Cre expression was detected in alveolar type II cells (A) and not BASCs (B). Only alveolar lung tumors were detected in *SPC^CreER^; p53^f/f^; Kras^LSL-G12D/+^* mice with leaky Cre activity (D) and BADJs retained normal architecture (C). Tumor cells expressed SPC (type II cell marker) and were highly proliferative (E), suggesting their origin from type II cells. By contrast, no obvious proliferation was observed at BADJs in these mice (F) consistent with their normal structure. Scale bar = 50 µm for panels in each row except (C, D), which is 100 µm.

We noticed spotty expression of CreER in some *SPC^CreER^*; *ROSA26^mTmG^* mice without tamoxifen injection (Fig. 1D, E). Interestingly, leaky CreER expression was only detected in alveolar type II cells (Fig. 3A) and not in BASCs or other lung cell types (Fig. 3B). We took advantage of this observation to further test the cells of origin of lung adenocarcinoma developed in *SPC^CreER/+^; p53^f/f^; Kras^LSL-G12D/+^* mice without tamoxifen injection. This would allow us to directly follow the fate of type II cells carrying *Kras/p53* mutations without the complication of BASCs.

We performed a careful immunohistochemical analysis of lungs collected from *SPC^CreER/+^; p53^f/f^; Kras^LSL-G12D/+^* mice without tamoxifen injection. These mice with leaky CreER expression without TM injection survived a few more months than those injected with TM. Similar to *SPC^CreER/+^; p53^f/f^; Kras^LSL-G12D/+^* mice injected with tamoxifen, invasive lung adenocarcinomas observed in *SPC^CreER/+^; p53^f/f^; Kras^LSL-G12D/+^* mice with leaky CreER expression localized to the alveoli (Fig. 3D, 3E). Tumor cells expressed type II cell marker (SPC) and were highly proliferative (Fig. 3E). BADJs, where BASCs reside, retained normal architecture (Fig. 3C) in *SPC^CreER/+^; p53^f/f^; Kras^LSL-G12D/+^* mice with leaky CreER expression and no obvious proliferation was observed at the BADJ (Fig. 3F). This provides additional evidence to support the notion that alveolar type II cells can be the initiating cells for lung adenocarcinoma.

### Activating *EGFR* in Murine SPC^+^ cells Led to Tumor Development in Alveoli

To further test the notion that alveolar type II cells can be the originating cells for NSCLC, we utilized our *SPC^rtTA^* mice to activate EGF signaling in SPC^+^ cells. We bred mice to produce *SPC^rtTA/+^; tetO-EGFR^L858R^* animals. The *tetO; EGFR^L858R^* mice harbor a transgene in which a mutant form of EGFR (EGFR^L858R^) is under the control of *tetO*; thus *EGFR^L858R^* expression can be induced upon doxycycline (dox) administration [23]. Importantly, the L858R mutation in EGFR is frequently found in human adenocarcinoma and is known to confer ligand-independent EGF signaling [36], [37]. *SPC^rtTA/+^; tetO-EGFR^L858R^* animals provide a means to regulate EGF signaling in cells that express rtTA.

**Figure 4:**
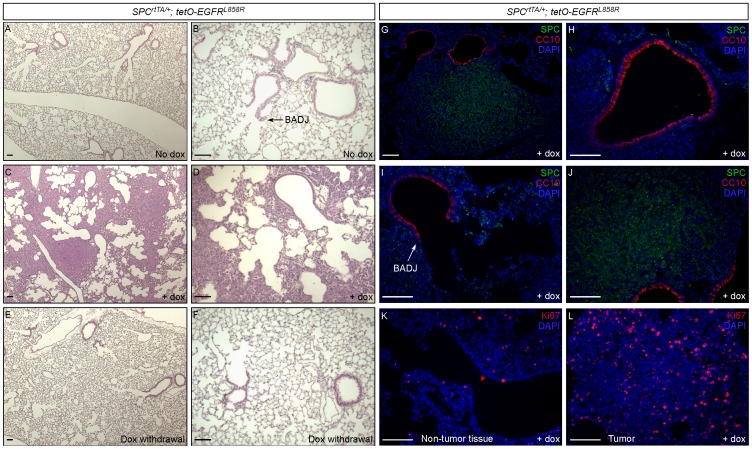
Induction of alveolar lung tumors in mice with activated EGF signaling in SPC^+^ cells. (A–F) Histology of lung sections from *SPC^rtTA/+^; tetO-EGFR^L858R^* adult mice. In the absence of dox chow, lung histology of *SPC^rtTA/+^; tetO-EGFR^L858R^* adult mice was similar to wild-type animals. Once fed with dox chow to activate EGF signaling, *SPC^rtTA/+^; tetO-EGFR^L858R^* mice developed alveolar tumors, but no abnormalities were detected in the airway or BADJ. Interestingly, upon dox withdrawal, tumors regressed and lung histology significantly improved. (G–L) Immunostaining of lung sections from *SPC^rtTA/+^; tetO-EGFR^L858R^* adult mice. Alveolar tumors in *SPC^rtTA/+^; tetO-EGFR^L858R^* adult mice fed with dox expressed SPC (G, J) but not CC10 (G, J). These tumor cells were highly proliferative as assessed by Ki67 staining (L), the entire field of which consists of tumor cells, compared to non-tumor tissues of the lung (K). No obvious hyperplasia was observed in the airway (H) or BADJ (I) in *SPC^rtTA/+^; tetO-EGFR^L858R^* adult mice with active EGFR signaling. Scale bar = 100 µm for each panel.

In the absence of dox, the lung morphology of *SPC^rtTA/+^; tetO-EGFR^L858R^* mice (Fig. 4A, 4B) was similar to that in wild-type mice. This suggests that even if low levels of *EGFR^L858R^* were expressed in some SPC^+^ cells in the absence of dox, they are insufficient to cause any phenotypes. Consistent with this, only spotty labeling of SPC^+^ cells by *R26R* or *ROSA26^mTmG^* reporters was observed in the absence of dox as described above (Fig. 1G, 1K), reflecting a low degree of leaky rtTA expression. By contrast, dox administration promoted rtTA binding to tetO and activated *EGFR^L858R^* in *SPC*-expressing cells. We found that after 12 weeks of continuous dox administration, *SPC^rtTA/+^; tetO-EGFR^L858R^* mice developed widespread tumors in the alveoli (Fig. 4C, 4D) but not in other parts of the lung, including the airway (Fig. 4H) and BADJ (Fig. 4I). No hyperplasia was detected in the airways or BADJs (Fig. 4C, 4D) and proliferating BASCs were rarely found. Tumor cells in *SPC^rtTA/+^; tetO-EGFR^L858R^* mice stained positive for SPC and negative for CC10 (Fig. 4G, 4J), and were highly proliferative (Fig. 4L) compared to non-tumor tissues (Fig. 4K). In conjunction with studies using *SPC^CreER^*, these results provided additional evidence to support the idea that NSCLC can be initiated in SPC^+^ type II cells. Interestingly, alveolar tumors regressed after dox withdrawal (Fig. 4E, 4F), suggesting that neoplasia is dependent on active EGFR signaling, consistent with previous reports [23]. This genetic system thus offers the advantage of studying the molecular process of tumor regression and resistance.

### Mouse Alveolar Tumors Induced by *Kras/p53* Mutations are Associated with Notch Pathway Activation

**Figure 5:**
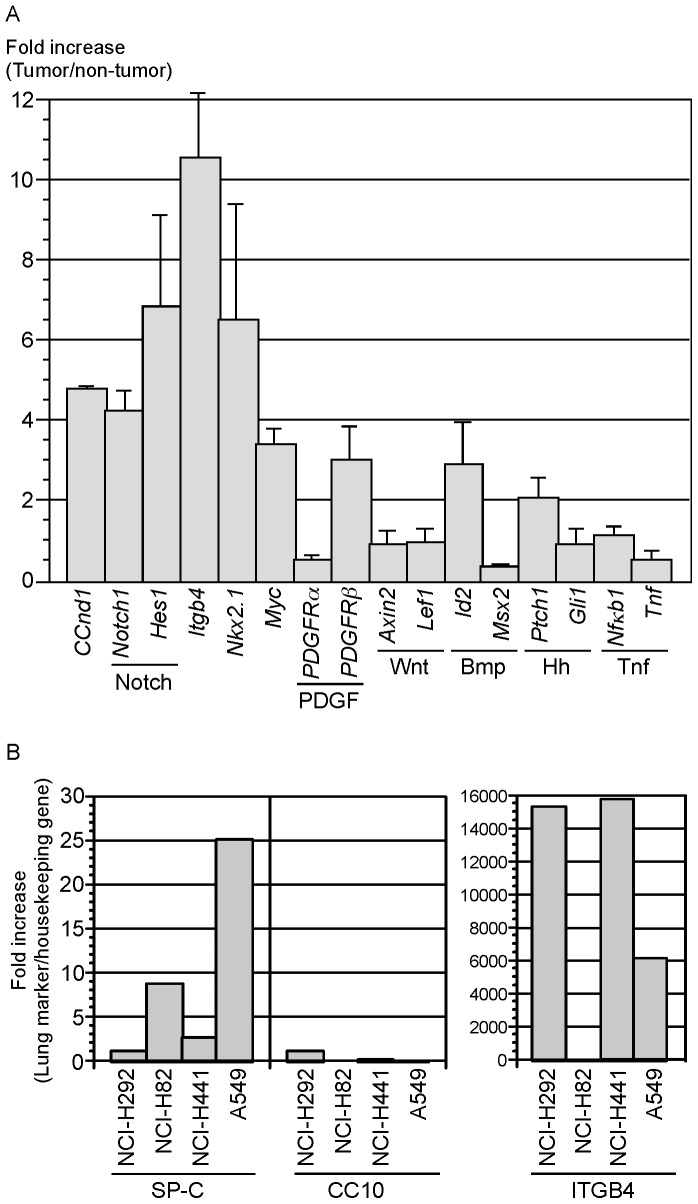
Analysis of major signaling pathways in mouse *Kras/p53* tumors and marker expression in human lung cancer cell lines. (A) Quantitative PCR (qPCR) analysis of RNA extracted from tumors and adjacent non-tumor tissues derived from *SPC^CreER/+^; p53^f/f^; Kras^LSL-G12D/+^* adult mice injected with tamoxifen. Target genes of each major signaling pathway were assessed for changes in their expression in tumor versus non-tumor tissues. The numbers represent fold of increase in tumor tissues. Multiple lung tumors from a single mouse and multiple mice were analyzed. *Ccnd1* (*cyclin D1*) was upregulated, consistent with increased Ki67 staining on tumor sections. Notch signaling was consistently upregulated in tumor versus non-tumor tissues whereas Platelet-derived growth factor (PDGF), Wnt, Bone morphogenetic protein (Bmp) and Hedgehog (Hh) pathways showed modest or no apparent changes in target gene expression. Expression levels of *Nkx2.1* and *Myc* were also significantly increased, similar to findings in human adenocarcinoma. Interestingly, expression of *Itgb4* (*integrin beta 4*) was also increased in tumor tissues. Future studies will determine whether Itgb4 plays a role in tumor development. (B) qPCR analysis of RNA extracted from representative human lung cancer cell lines, including NCI-H292 (pulmonary mucoepidermoid carcinoma), NCI-H82 (small cell lung cancer), NCI-H441 (papillary adenocarcinoma of the lung) and NCI-A549 (lung adenocarcinoma). The numbers represent fold of increase in marker expression relative to housekeeping genes. *SPC* expression was upregulated in the A549 cell line. *ITGB4* (*integrin beta 4*) was also highly expressed in non-small cell lung cancer cell lines compared to small-cell lung cancer cell lines.

As a first step toward understanding the molecular mechanisms that underlie tumor development and confer tumor properties, we investigated whether major signaling pathways were perturbed in lung tumors developed in *SPC^CreER/+^; p53^f/f^; Kras^LSL-G12D/+^* mice. We extracted RNA from dissected tumors and adjacent non-tumor tissues and performed qPCR analysis to examine the expression of target genes for major signaling pathways. Interestingly, we found that Notch signaling was consistently upregulated in tumor versus non-tumor tissues (Fig. 5A). By contrast, several other major signaling pathways, including the Platelet-derived growth factor (PDGF), Wnt, Bone morphogenetic protein (Bmp) and Hedgehog (Hh) pathways, showed modest or no apparent changes in target gene expression in tumor tissues compared to non-tumor tissues (Fig. 5A). Interestingly, expression of Itgb4 (integrin beta 4) was also increased in mouse lung tumor tissues (Fig. 5A) as well as in human non-small cell lung cancer cell lines (Fig. 5B). Notch signaling has been associated with survival of lung adenocarcinoma cells *in vitro* [38] and tumor metastasis [39]. Our findings suggest that activated Notch signaling in response to *Kras/p53* mutations could be involved in tumor development or confer tumor properties. The genetic system we developed provides an ideal setting to isolate tumor cells at different stages of tumor development and ask how Notch signaling controls tumor initiation, progression and invasion/metastasis. These studies will provide mechanistic insight into the roles of major signaling pathways in lung tumor development.

## Discussion

Through the development of new genetic tools in mice, we provide strong evidence to support the notion that human NSCLC can originate from alveolar type II cells. Our results confirm and extend the findings in a recent report [40], which primarily analyzed mice carrying *Kras* mutations. No evidence was presented in the published article [40] to demonstrate tumor invasion or metastasis. It is worth noting that earlier reports using adenoviral-Cre to infect lung epithelium globally showed that mice carrying the *Kras* mutation and one loss-of-function allele of *p53* in the lung exhibited phenotypes similar to mice carrying the *Kras* mutation alone [41]. This suggests that loss of both copies of *p53* is critical for invasive tumors to develop. Our experimental setting enabled us to achieve simultaneous *Kras* activation and *p53* ablation. As a result, tumor invasiveness was observed in *Kras/p53* mutants but not in mice carrying activated *Kras* alone under our experimental conditions. Importantly, taking advantage of the leaky expression of our *SPC^CreER^* line only in alveolar type II cells but not in BASCs, we were able to attribute lung adenocarcinoma developed in these mice to type II cells and not BASCs. Taken together, these new findings strongly support the idea that development of lung adenocarcinoma (beyond adenoma or carcinoma *in situ*) originates from SPC^+^ alveolar type II cells.

A previous report [23] relied on a *CCSP-rtTA* transgene to investigate how EGFR mutations in different lung cell types could influence tumor development. The *CCSP-rtTA* transgene utilizes a short fragment of the rat *CCSP* promoter to drive rtTA expression. The short rat *CCSP* promoter does not recapitulate mouse *CCSP* (*CC10*) expression and displays significant leaky expression in SPC-expressing cells, raising the possibility that it is expressed in BASCs. How EGFR mutations affect BASC proliferation/transformation was not addressed in previous work [23]. Our *SPC-rtTA* mouse line in which rtTA is under the control of the endogenous *SPC* promoter provides the first mouse model to assess the effects of EGFR mutations on transforming SPC^+^ type II cells.

While our results suggest that NSCLC could initiate in type II cells, the exact nature of tumor-initiating cells requires further investigation. Without tracing the early events at the molecular level, it is not possible to decipher how differentiated type II cells that carry oncogenic mutations undergo progressive transformation. It is possible that all SPC-expressing type II cells share a similar potential of being transformed. Alternatively, tumors may only initiate in a small population of uncharacterized type II cells, for instance, those expressing low levels of SPC and presumably possessing proliferative potential in various conditions [42] or those residing at specific anatomical locations. Without markers currently available to define subpopulations of type II cells, it is not possible to assess the transformation potential of distinct subpopulations of type II cells, if they do exist. It is also uncertain how the transformation potential of type II cells is compared to other lung epithelial cell types such as Clara cells, BASCs, ciliated cells, goblet cells and type I cells under various genetic perturbations. Perhaps, distinct cell types are susceptible to specific insults and a systematic investigation is required to delineate the molecular basis of proliferative potential among distinct lung cell types or populations. These studies would shed light on the source of heterogeneity or subtypes of NSCLC, the very properties of which could originate from distinct cell types carrying different mutation profiles. Finally, we cannot rule out the possibility that cells with altered p53 and Kras activities may exert non-cell autonomous effects on neighboring cells and induce tumor development in these cells containing wild-type *p53* and *Kras*.

We found some degree of leakiness present in both *SPC^CreER^* and *SPC^rtTA^* mouse strains that we used to manipulate gene activity in type II cells. In particular, more sensitive reporter lines (*e.g.*, *ROSA26^mTmG^*) appear to detect even very low levels of leaky expression. In some cases, variations in background expression among individual mice were also noted. Consistent with these findings, when leaky CreER expression reached a critical threshold in *SPC^CreER/+^; Kras^LSL-G12D/+^* or *SPC^CreER/+^; p53^f/f^; Kras^LSL-G12D/+^* mice, cell hyperplasia and even tumor development was observed without TM administration albeit at a lower frequency than in mice injected with TM. Interestingly, even in mice in which leaky CreER expression led to tumor development, tumors were never detected at the BADJ. By contrast, *SPC^rtTA/+^; tetO-EGFR^L858R^* mice without dox do not develop hyperplasia or tumors. This probably reflects differential sensitivities of type II cells to diverse perturbations.

Given that *SPC^CreER^* confers the ability to manipulate gene activity in BASCs, it is somewhat surprising that hyperplasia at the BADJ was not observed in *SPC^CreER/+^; Kras^LSL-G12D/+^* or *SPC^CreER/+^; p53^f/f^; Kras^LSL-G12D/+^* mice when CreER is activated by TM. This raises the possibility that BASCs are not as sensitive to Kras/p53 perturbation as other SPC-expressing cells. Similarly, active EGF signaling in SPC-expressing cells only led to alveolar tumor development and not BASC hyperplasia or neoplasia. Since BASCs express both SPC and CC10, it would be interesting to compare the transformation potential between BASCs and Clara cells. Whereas our results do not provide evidence to support a role for BASCs in tumor development, it is conceivable that additional genetic perturbation not utilized in this study may be required to transform BASCs. It is also possible that a longer time period is needed for tumors to develop in BASCs. This is consistent with the observation that human lung tumors usually develop after many years or even decades of repeated epithelial injury such as from cigarette smoking. Though BASC proliferation is rare in our experimental setting, we cannot formally rule out the possibility that BASCs can be transformed, expanded and differentiated into type II-like cells. Lineage-tracing studies in which *Kras/p53* mutant BASCs and type II cells are labeled and followed during early tumor development will provide new insight into this important issue.

Human NSCLC is a heterogeneous group of diseases that share common histology but carry different mutations and perhaps even originate from different cell types [43]. For instance, some NSCLC cell lines express markers of alveolar type II cells (Fig. 5B). By contrast, the mouse models we developed likely mimic only a subset of human lung cancer. A key feature of the system is its ability to mark alveolar type II cells and trace the early events of tumor development. Equally important is the opportunity that this system offers to isolate transformed type II cells induced by different genetic perturbations at various stages of progression for whole-genome analysis (including RNA-Seq and Exome sequencing). These studies will yield key insight into the molecular mechanisms of transformation. Such analysis will also provide candidates for early diagnosis and targeted therapies of NSCLC [44]. We speculate that our mouse model offers a defined genetic system with a shorter time frame of tumor development in which fewer mutations are accumulated in the process of transformation compared to human lung cancer. Employing mouse models would facilitate the identification of relevant candidates for diagnosis and treatment through comparative genomic analysis between human and mouse tumors.

## Materials and Methods

### Mice

All animal work was carried out in strict accordance with the recommendations in the Guide for the Care and Use of Laboratory Animals of the National Institutes of Health. The protocol was approved by the Institutional Animal Care and Use Committee at the University of California, San Francisco.

To produce the *SPC^CreER^* mouse line, the translational start ATG in the mouse *SPC* genomic locus was replaced with a CreER-FRT-PGK-Neo-FRT cassette through gene targeting in E14 embryonic stem (ES) cells. The FRT-PGK-Neo-FRT cassette was subsequently removed by crosses with *FLPe* mice [45].

The *SPC^rtTA^* mouse line harbors an engineered bacterial artificial chromosome (BAC) in which rtTAtPA was placed at the translational start ATG of the *SPC* genomic locus and tetO-Cre was inserted at the translational start ATG of the *Nudt18* genomic locus. To generate the *SPC^rtTA^* BAC, an rtTAtPA targeting construct containing homologous arms to the SPC genomic locus was introduced by the GalK cloning system [46] into a mouse BAC clone (RP23-247J9; CHORI). RP23-247J9 spans the *SPC* and *Nudt18* genomic regions with the *SPC* gene centering on RP23-247J9. After screening correctly targeted clones at the *SPC* locus, the recombined BAC underwent a second recombination to introduce tetO (TRE-Tight)-Cre to the *Nudt18* locus. The *Nudt18* gene is located ∼57 kb upstream of the translational start ATG of *SPC*. BAC DNA was linearized with *PI-SceI* and introduced into CD-1 zygotes by pronuclear injection to produce transgenic mice.

*p53* floxed (FVB.129-*Trp53^tm1Brn^*), *Kras* ^LSL-G12D^ (B6.129-*Kras^tm4Tyj^*) and *tetO-EGFR^L858R^* (B6; CBA-Tg(tetO-EGFR*L858R)56Hev) mice were obtained from the NCI Mouse Repository. *ROSA26^mTmG^* (Stock *Gt(ROSA)26Sor^tm4(ACTB-tdTomato,-EGFP)Luo^/*J) and *R26R* (B6; 129S4-*Gt(ROSA)26Sor^tm1Sor^*/J) mice were obtained from Jackson Laboratory.

### Tamoxifen Administration

Tamoxifen (TM) (Sigma) was dissolved in Mazola corn oil to make a stock solution of 50 mg/ml. TM (0.25 mg/g body weight) was injected into adult mice daily for four to six days. Lungs were collected for analysis at indicated time points.

### Histology, Immunohistochemistry and β-galactosidase Staining

Mouse lungs were inflated with 1 ml of 2% paraformaldehyde (PFA) and fixed for 4 hrs-overnight at 4°C in 2% PFA. After dehydration and processing, lungs were embedded in paraffin for sectioning and immunohistochemistry. All the tissues collected were sectioned at 6 µm thickness for histological analysis. Paraffin sections were stained with the following primary antibodies: chick anti-GFP (1∶200, Abcam), goat anti-CC10 (1∶200, Santa Cruz), rabbit anti-proSP-C (1∶400, Millipore), hamster anti-T1α (1∶200, Developmental Studies Hybridoma Bank), mouse anti-Ki67 (1∶200, BD) and goat anti-Sox2 (1∶200, Santa Cruz). Secondary antibodies and conjugates used include: donkey anti-chick DyLight 488 (1∶1,000; Jackson ImmunoResearch Laboratories), donkey anti-rabbit Alexa Fluor 594 (1∶1,000; Life Technologies), donkey anti-goat Alexa Fluor 594 or 647 (1∶1,000; Life Technologies), donkey anti-mouse Alexa Fluor 594 or 647 (1∶1,000; Life Technologies), and DAPI (1∶10,000; Sigma). For biotinylated secondary antibodies (goat anti-rabbit, 1∶1,000, donkey anti-goat, 1∶1,000 and horse anti-mouse, 1∶1,000, Jackson ImmunoResearch Laboratories), the signal was detected using HRP-conjugated streptavidin (1∶1,000, Vector Laboratories) in combination with either the chromogenic substrate DAB (Vector Laboratories) or fluorogenic substrate Alexa Fluor 594 tyramide (1∶100, TSA kit, Perkin-Elmer). Antibodies against Ki67 may require the biotin-streptavidin amplification step for optimal signal detection. Fluorescent images were acquired using a SPOT 2.3 CCD camera connected to a Nikon E1000 epifluorescence microscope. Adjustment of RGB histograms and channel merges were performed using SPOT Advanced software or ImageJ. Confocal images were captured on a Leica laser-scanning confocal microscope. Multiple optical sections were obtained to distinguish cell boundaries. For X-gal staining, lungs were inflated with 1 ml of 1% PFA, 0.2% gluteraldehyde and fixed 1 hr at 4°C in 1% PFA and 0.2% gluteraldehyde. To detect β-galactosidase activity, whole lungs or frozen sections (10 µm) were stained in X-gal staining solution following standard procedures.

### Analysis of Target Gene Expression for Major Signaling Pathways in Mouse Lung Tumors and Lung Marker Expression in Human Lung Cancer Cell Lines

Tumor and adjacent non-tumor tissues were microdissected from the mouse lungs. Human lung cancer cell lines were purchased from ATCC. Total RNA was extracted using Trizol (Invitrogen) and RNeasy Mini Kit (Qiagen) and subsequently reverse-transcribed to cDNA using the SuperScript Kit (Invitrogen). Quantitative PCR (qPCR) was carried out on the ABI Prism 7900HT Sequence Detection System.

The following primers for mouse genes were used: *Ccnd1* F: 5′GCAGAAGGAGATTGTGCCATCC3′; *CCnd1* R: 5′AGGAAGCGGTCCAGGTAGTTCA3′; *Notch1* F: 5′GCTGCCTCTTTGATGGCTTCGA 3′; *Notch1* R: 5′CACATTCGGCACTGTTACAGCC3′; *Hes1* F: 5′GGAAATGACTGTGAAGCACCTCC3′; *Hes1* R: 5′GAAGCGGGTCACCTCGTTCATG3′; *Itgb4* F: 5′ACACAAGCTCCAGCAGACGAAG3′; *Itgb4* R: 5′TCCACCTGCTTCTCTGTCAGCT3′; *Nkx2.1* F: 5′CAGGACACCATGCGGAACAGC3′; *Nkx2.1* R: 5′GCCATGTTCTTGCTCACGTCCC3′; *Myc* F: 5′TCGCTGCTGTCCTCCGAGTCC3′; *Myc* R: 5′GGTTTGCCTCTTCTCCACAGAC3′; *PDGFR*α F: 5′GCAGTTGCCTTACGACTCCAGA3′; *PDGFR*α R: 5′GGTTTGAGCATCTTCACAGCCAC3′; *PDGFRβ* F: 5′ACTACATCTCCAAAGGCAGCACCT3′; *PDGFRβ* R: 5′TGTAGAACTGGTCGTTCATGGGCA3′; *Axin2* F: 5′ATGGAGTCCCTCCTTACCGCAT3′; *Axin2* R: 5′GTTCCACAGGCGTCATCTCCTT3′; *Lef1* F: 5′ACTGTCAGGCGACACTTCCATG3′; *Lef1* R: 5′GTGCTCCTGTTTGACCTGAGGT3′; *Id2* F: 5′TCACCAGAGACCTGGACAGAAC3′; *Id2* R: 5′TGCTATCATTCGACATAAGCTCAG3′; *Msx2* F: 5′AAGACGGAGCACCGTGGATACA3′; *Msx2* R: 5′CGGTTGGTCTTGTGTTTCCTCAG3′; *Ptch1* F: 5′GCGCAGTCTTCCTCCTGAAC3′; *Ptch1* R: 5′GAGGCCCATCATGCCAAAG3′; *Ptch1* probe: 5′CCTGGACGGCCGGGATCATT3′; *Gli1* F: 5′GACTGCCGCTGGGATGGTTGCAG3′; *Gli1* R: 5′GCGTGAATAGGACTTCCGACAGCCT3′; *Gli1* probe: 5′ATTCCCAGGAGCAGCTGGTGCACCACAT3′; *Nfκb1* F: 5′ GCTGCCAAAGAAGGACACGACA3′; *Nfκb1* R: 5′GGCAGGCTATTGCTCATCACAG3′; *Tnf* F: 5′GGTGCCTATGTCTCAGCCTCTT3′; *Tnf* R: 5′GCCATAGAACTGATGAGAGGGAG3′; *β-actin* F: 5′CTGCCTGACGGCCAGGT3′; *β-actin* R: 5′TGGAAAAGAGCCTCAGGGC3′; *β-actin* probe: 5′TCGGAACCGCTCGTTGCCAATAGT 3′; *Gapdh* F: 5′AGGTTGTCTCCTGCGACTTCA3′; *Gapdh* R: 5′CCAGGAAATGAGCTTGACAAAGTT3′.

The following primers for human genes were used: *SFTPC* (SP-C) F: 5′AGCCAGAAACACACGGAGATGGTT3′; *SFTPC* (SP-C) R: 5′ ATCTTCATGATGTAGCAGCAGGTGCC3′; *SCGB1A1* (*CC10*) F: 5′TCCTCCACCATGAAACTCGCTGT3′; *SCGB1A1* (*CC10*) R: 5′TTCCATGGCAGCCTCATAACTGGA3′; *GAPDH* F: 5′AAGGTGAAGGTCGGAGTC3′; *GAPDH* R: 5′GATTTTGGAGGGATCTCG3′. House keeping genes including β*-actin* and *Gapdh* were used as internal controls for qPCR.
